# Neuroticism Predicts a Long-Term PTSD After Earthquake Trauma: The Moderating Effects of Personality

**DOI:** 10.3389/fpsyt.2019.00657

**Published:** 2019-09-20

**Authors:** Qianlan Yin, Lili Wu, Xiaoqian Yu, Weizhi Liu

**Affiliations:** ^1^The Emotion & Cognition Lab, Faculty of Psychology and Mental Health, Second Military Medical University, Shanghai, China; ^2^Department of Psychology, University of South Florida, Tampa, FL, United States

**Keywords:** traumatic experience, posttraumatic stress disorder, personality, neuroticism, Wenchuan earthquake

## Abstract

**Background:** The heartache from the devastating 8.0 magnitude Wenchuan earthquake, which killed nearly 90,000 people in western China, is still felt despite the large-scale recovery and reconstruction of the affected areas. This study investigated the relationships of earthquake-trauma exposures and personality with posttraumatic stress disorder (PTSD), to identify the long-term consequences of the Wenchuan earthquake on the survivors and the risk factors related to chronic PTSD. We hope the findings can contribute to developing new health care prevention and interventions for the survivors.

**Methods:** We collected a sample of 490 people over 3 years after the Wenchuan earthquake, using questionnaires about demographic information and the traumatic experience in earthquake, the Impact of Event Scale-Revised (IES-R), and the Eysenck Personality Questionnaires (EPQ), to find the consequences of the Wenchuan earthquake on the survivors and the potential factors related to the long-term morbidity of PTSD.

**Result:** Traumatic experiences, such as witnessing someone being seriously injured, having your house seriously damaged, and having close relatives severely injured, were associated with developing PTSD. Personality measured by EPQ was also closely related to PTSD. Regression analyses indicated that a potential linear model characterized the relationship between PTSD, neuroticism and psychoticism, yet extraversion/introversion were not significant factors. In the multivariate logistic regression, neuroticism (a continuous variable measured by EPQ) was of more significance in predicting the morbidity of long-term PTSD, compared with other variables [odds ratio (OR) = 1.113, 95% confidence interval (CI) = 1.081-1.146, Wald Value = 50.467, P < 0.001]. The final path diagram built a model indicated the moderating role of personality in the relationship between traumatic experiences and PTSD (CMIN/DF = 2.324, P < 0.001; CFI = 0.879 < 0.8; RMSEA = 0.05 < 0.08).

**Conclusion:** This study demonstrated the role of personality traits and subjective exposure experiences regarding the vulnerability associated with PTSD after earthquake. Among all personality traits, neuroticism is considered a vulnerability factor of PTSD, and other personality traits also moderate the effects of traumatic experiences associated with PTSD. These findings might be useful for psychologists to develop intervention strategies for people suffered natural disasters, and to help individuals with PTSD to heal fully.

## Introduction

Exposure to a disaster can trigger a wide range of psychological consequences including severe psychological distress, immense physical devastation and emotional suffering coupled with a terrible feeling of loss, which could severely affect individuals’ psychosocial functioning and quality of life (1). On May 12, 2008, a powerful 8.0-magnitude earthquake struck Wenchuan, a county located in the Sichuan province of China. In addition to the vast amount of people who lost their lives, the earthquake left nearly 1,486,407 people homeless, with over 46.25 million people living in the affected area (2). The psychological impact remains even ten years following the earthquake. In fact, the mental recovery could take at least 10 years, and might take up to 20 years (3). Given its enduring negative impact, abundant research has been conducted to address the mental and physical recovery need for survivors (4).

Posttraumatic stress disorder (PTSD), a trauma-related mental disorder, has been found to be the most common psychiatric disorder among earthquake survivors (5, 6). PTSD impairs one’s physical or psychological integrity with symptoms such as intrusive unwanted recollections of the event, attempts to get these recollections out of consciousness, elevated levels of arousal, and negative changes in the cognition and mind (6). PTSD trajectories in previous trauma studies include ‘resistance’ (minimal or no symptoms over time), ‘chronic dysfunction’ (moderate or severe symptoms over time), ‘delayed dysfunction’ (initially minimal/no symptoms followed by elevated symptoms), ‘recovery’ (initially moderate/severe symptoms followed by a gradual return to pre-trauma functioning) and ‘relapsing/remitting’ (symptoms displaying a cyclical course) (7). The findings of this large body of research indicate that the Wenchuan earthquake has had devastating and long-lasting impacts for survivors (8–11). An et al. (12) reported longitudinal PTSD symptoms in adolescents following the 2008 Wenchuan Earthquake at three time points: 1 year, 1.5 years and 2 years after the earthquake, which suggested a slight decreasing trend 2 years after the earthquake. The statistics in a 10-year review show that the prevalence rate of PTSD in Wenchuan earthquake community studies was much higher than 23.66% in the first 3 months after the earthquake and was still near 20% after 12 months (13). PTSD prevalence rates at 6, 12, 18, and 24 months were 21.0, 23.3, 13.5 and 14.7%, respectively, in 1573 adolescent survivors, reported in Wang and Gao (14). Moreover, one year after the Wenchuan earthquake, 14.1% of college students were diagnosed with PTSD (15). The prevalence of PTSD was 8.8% among survivors from the hard-hit areas 3 years after the Wenchuan earthquake (16). A recent study also revealed that 11.8% of 1369 respondents from two severely affected areas by Wenchuan earthquake had PTSD symptoms 8 years post-disaster (17). However, risk factors related to long-term PTSD development over a 6-month period has not been well-studied even though there is a growing consensus that great individual differences exist in terms of posttrauma long-term responses (18, 19). Therefore, a better understanding of its risk factors can contribute to both prevention and treatment for long-term PTSD.

Studies from the past decade have focused on PTSD and potential predictors of PTSD symptoms after disaster. Liang et al. (13) concluded that PTSD after an earthquake has been associated with a range of risk factors, including sociodemographic factors, trauma exposure characteristics, post-disaster cognitive and emotional states, and social support. Referring to the review in which the relative contribution of the factors were examined (all factors were entered into the analyses simultaneously) (20), personality emerged as a robust independent predictor, suggesting that developing PTSD could be due to differences in personality traits, which directly impacts responses to external stimuli (e.g., disaster) through cognitive processes, coping strategies, and maybe interaction with social support processes. McFarlane (21, 22) reported that introversion and neuroticism were associated with the chronic development of post-traumatic stress symptoms among fire-fighters. Wen et al. (16) also reported rates of PTSD among students in the Wenchuan earthquake, showing that individuals high in introversion and neuroticism were more susceptible to PTSD. Moreover, several prospective investigations have reported that neuroticism was positively associated with the prevalence of PTSD symptoms (23–25). Neuroticism, characterized by a negative emotional response to threat, frustration or loss, overlaps specific aspects with arousal symptoms. This overlap content consisted of the longitudinal trajectories of PTSD symptoms, explaining why neuroticism is considered a vulnerability factor of PTSD. Therefore, personality factors, as long-standing traits existing before and after, could predict an individual’s vulnerability to develop PTSD and we suggested it to be a potential vulnerability factor for developing long-term PTSD after the Wenchuan earthquake. In addition, trauma factors of an earthquake associated with the trauma severity should also be taken into account as the rate of PTSD can vary with severity of the event and the duration of trauma exposure, which was one of the most important post-disaster stressors for PTSD (26, 27). For instance, Chen and Xu et al. (28) investigated the role of personality traits and subjective exposure experiences in PTSD among 20,749 children who experienced the Wenchuan earthquake and found that, besides neuroticism, subjective exposure experiences are also risk factors for PTSD. Furthermore, Guo et al. (29) highlighted the interacted effect of trauma exposure and trait neuroticism on PTSD symptoms among adolescents exposed to a pipeline explosion. Although it has been acknowledged that earthquake exposure is associated with PTSD, it is yet unclear as to how it affects the development of PTSD in the long-term, neither is the intertwined effect with personality. Referring to research about PTSD occurring after the maltreatment of children, traumatic experience was the strongest predictor of PTSD, while personality modified the impact of contextual factors. However, the majority of existing studies were cross-sectional and assessed PTSD at a single time point. Most people develop PTSD symptoms within 3 months following the traumatic event, while some people do not notice any symptoms until years later. Only a few studies have examined the trajectory of PTSD symptoms among the Wenchuan earthquake adolescent survivors and acknowledged the effect of traumatic experiences, but they still lacked explanations for the role played by personality on chronic PTSD (8).

Given that long-term PTSD is the most common psychological disorder after an earthquake, the current study aimed to investigate the predictive factors of PTSD about 3 years after the earthquake, and to examine the interplay among the risk factors relating to the genesis of PTSD. We conducted a comparative analysis among a large sample of the Wenchuan earthquake survivors to examine the relationship between personality traits and traumatic experience on PTSD while controlling for other risk factors. According to the estimation, these survivors of the Wenchuan earthquake included a large amount of adolescents, who are generally more vulnerable when they are exposed to trauma and, thus, they often exhibit traumatic reactions following disasters and suffer from longer mental injuries (30). Therefore, this study was undertaken primarily to focus on this vulnerable group and to elucidate traumatic exposure experience, to highlight personality traits such as neuroticism as potential risk factors for PTSD, and to show that neuroticism could moderate the effect of traumatic experience on PTSD. Differing from previous studies, we used the Eysenck Personality Questionnaire to measure the survivors’ post-trauma personalities for an adequate classification accuracy, sensitivity, and specificity. A self-adapted traumatic experience questionnaire was designed for the earthquake survivors to evaluate the degree of traumatic exposure. We hope to explore the interactive effect of personality traits and traumatic experience on PTSD among Wenchuan earthquake survivors from a long-term perspective, and to contribute to the development of preventive interventions and treatment programs for PTSD. Cultural differences from previous studies conducted in foreign contexts would also be discussed.

## Method

### Participants

A cross-sectional survey was conducted in earthquake affected areas of Sichuan province through the multistage cluster sampling method. The data were obtained from two sample surveys between July 2010 and May 2011. First, we randomly selected two cities (Sichuan-Aba Tibetan & Qiang autonomous prefecture and Mianyan Qiang autonomous prefecture) from the hardest-hit areas. In these two prefectures, Maoxian and Beichuan were selected *via* a simple random sampling method. As the disaster area is relatively underdeveloped, schools in each city are mainly concentrated in one area, where the sampling collection is suited to be conducted. Hence, with a stratified sampling method, participants were recruited from junior and senior high schools at the unit of class. In each grade, we randomly selected half classes of this grade in the school. Background information included traumatic history about the participants which was collected to evaluate if they were eligible for the following survey according the following criteria.

1. The Wenchuan earthquake experience is the only traumatic experience to them; no experience of other types of trauma such as losing a family member, divorcing, or car accident.
2. Can read and write normally.

Finally, we distributed a total of 490 questionnaires and excluded 33 questionnaires that were not completed by participants from the earthquake-affected area, resulting in 457 valid questionnaires (93.3%). The error and missing rate of the data was lower than 10% and replaced by the mean value. Participants were equally capable to complete the questionnaires since they were middle school and high school students. Participants’ age ranged from 12 to 20, which is higher than the average age of the usual middle and high school students. This is due to late attendance at school among minorities because of financial or cultural reasons. The criteria for the target group are listed below:

### Measures

The surveys include (1) demographic measures such as gender, age, and education level (grade); (2) traumatic experience in earthquake; (3) the Impact of Event Scale-Revised (IES-R) and (4) the Eysenck Personality Questionnaires (EPQ).

### Questionnaires About the Traumatic Experience in Earthquake

The questionnaire includes 10 questions:
E1- Having been in serious danger;
E2- Having witnessed someone being seriously injured;
E3- Having witnessed people dying;
E4-Having close relatives (parents, sisters, brothers, grandparents and so on) injury severely;
E5-Having close relatives (parents, sisters, brothers, grandparents and so on) dead in the earthquake;
E6- Having good friends injured severely or dead;
E7- Having been your body hurt;
E8- Having your house damaged seriously;
E9- Having witnessed a tragic scene after the earthquake;
E10- Having felt scared about the earthquake.

The first 9 questions were coded into yes/no items, while the 10^th^ question was measured on a Likert scale, ranging from 1 (slightly) to 3 (intense).

### The Impact of Event Scale–Revised (IES-R)

The Impact of Event Scale-Revised (IES-R) was a 22-item commonly used, psychometrically sound self-report questionnaire to measure PTSD symptomatology (31–33). Each item can be scored from 0 (no problems) to 4 (frequent problems), and the cut-off score is suggested to be 40 (34). A score larger than 40 can be used to estimate the severity of PTSD symptoms, with higher IES-R scores indicating more severe PTSD symptoms (33, 35, 36). In our study, the Cronbach’s α as a reliability coefficient was 0.79-0.91. Test-retest reliability ranged from 0.51 to 0.94 (31). The item-total correlations were significant (r = 0.44-0.68, α < 0.001). The validity analysis showed that the coefficient of the IES-R total score was 0.90. The Chinese version of the IES-R is comparable to the original English version and with satisfactory psychometric properties (37).

### Eysenck Personality Questionnaires

The Eysenck Personality Questionnaire measures traits of psychoticism, extroversion, neuroticism, and lie scale. It has the needed quality of psychological scale and has been employed at home and aboard for at least 40 years (38). The Chinese version of the Eysenck Personality Questionnaire (39) was used to assess neuroticism (N), extraversion (E), and psychoticism (P). In our sample, Cronbach’s α for neuroticism and extraversion scales were 0.85 and 0.69, respectively. The negative correlation between the neuroticism with extraversion subscale was 0.217 (p < 0.001).

### Procedure

This study was approved by the ethics committees of the Second Military Medical University in Shanghai. After obtaining the approval from the dean and teachers in the schools, informed written consent was obtained from adolescents’ guardians, together with oral approvals from participants. According to the Declaration of Helsinki, a complete survey description was first presented to the participants. Five experienced researchers with training in epidemiology and unified training went from class to class to distribute and explain the purpose of the survey. Each student received 10 CNY in exchange of taking the survey, and each researcher was paid 200 CNY. After surveys were collected, researchers would do a site assessment.

### Statistical Analysis

Considering the different variable attributes, demographic and earthquake exposure data between PTSD and non-PTSD groups were compared by χ^2^ test. Linear regression analysis and t-tests were conducted to examine the relationship between personality and PTSD, both were entered as continual variables. Multivariable logistic regression was used to analyze the associated variables and their prediction to the morbidity of PTSD. The entry and removal criteria for the variables in the forward stepwise analysis were 0.05 and 0.10, respectively. Odds ratios (ORs) and their 95% confidence intervals (CIs) were presented to show the association. P < .05 was considered statistically significant and the value of Wald implied the predictor dependability. Data were analyzed in SPSS version 17.0 (SPSS Inc, Chicago, IL). Structural equation model was built and analyzed in AMOs 21.0 in the way purposed by Ping’s studies (40). The estimation methods were applied for the nonlinear latent variables, regression analyses, subgroup analyses, and structural equation analyses. The technique specifies these variables with single indicants, and the loading and error terms which can be specified as constants in the structural model. We added a moderator variable whose value was the product of the original two variables’ (personality and traumatic experience) standard scores and then tested whether the pathways among the three variables to the depended variable were significant. The moderating effect was proved to be existed if the P value of the pathways lower than 0.05. The maximum likelihood (ML) estimation method was employed to estimate the parameters of the latent constructs. The results suggested an acceptable fit: CMIN/DF = 2.243, P < 0.001; CFI = 0.897 > 0.8; NFI = 0.810 > 0.8; IFI = 0.885 > 0.8; RMSEA = 0.05 < 0.08. All the lambda parameters were significant (p < 0.01) and lower than 0.05; hence supporting convergent validity. As a single-method study, we considered the common method bias (CMB) in this model, and the result of the CMB model showed CMIN/DF = 2.297, P < 0.001; CFI = 0.876; NFI = 0.810; IFI = 0.883; RMSEA = 0.052, which was not better than the former. Therefore, the effects of an unmeasured latent methods factor were not significantly on the moderation-effect model.

## Results

### The Basic Demographic Information

The 457 valid questionnaires were from 266 males (58.2%) and 191 females (41.8%) with a mean age of 15.47 ± 2.28. The subjects comprised 252 junior high school students (55.1%) and 205 senior high school students (44.9%). They are from different ethnic groups. 382 (83.2%) Qiang, 34 (7.4%) Hui, 24 (5.3%) Han, and other took up 3.7% (n = 17). We divided the participants into a PTSD group (n = 104) and a non-PTSD group (n = 353) with the measure of IES-R and the score 40 as a cut-off, for studying effects of the traumatic experience and then performed the chi-square test in the PTSD group on the demographic data and traumatic experience.

### Traumatic Experience in People With PTSD

The results illustrated in the **Table 1** shows that there was no discrepancy in the possibility with PTSD for people regardless of gender and ethnicity (gender:χ^2^ = 0.11, p = 0.916; age:χ2 = 2.131, p = 0.546, the educational level:χ^2^ = 7.673, p = 0.06, at the standard of α = 0.05). As for the traumatic experience, the answer for E2, E3, E4, E6, E8, E9, and E10 (χ^2^_E2_ = 24.498 _P_ = 0.001 < 0.05; χ^2^_E3_ = 10.88 _P_ = 0.001 < 0.05; χ^2^_E4_ = 9.641 _P_ = 0.002 < 0.05; χ^2^_E6_ = 6.221 _P_ = 0.013 < 0.05; χ^2^_E8_ = 10.175 _P_ = 0.001 < 0.05; χ^2^_E9_ = 10.013 _P_ = 0.002 < 0.05; χ^2^_E10_ = 13.16 _P_ = 0.001 < 0.05) had closely relationship with PTSD, while no significant relation with other exposures (P > 0.05). Hence, a significantly higher prevalence of PTSD symptoms was found among people who had witnessed someone, either relatives or good friends, who were seriously injured or dying, who had house damaged seriously, who had witnessed a tragic scene after the earthquake, and who had felt scared.

**Table 1:** Symptoms of PTSD with demographics and earthquake-related experiences among junior and senior high school students following the Wenchuan earthquake, China (n = 457).

Variable	PTSD (n = 104, 22.8%)			
	Percentage	N	χ2	p
Demographics				
Gender			0.011	0.916
Male	22.51%	43		
Female	22.93%	61		
Ethnicity			2.131	0.546
Qiang	23.30%	89		
Hui	14.71%	5		
Han	29.17%	7		
Others	17.65%	3		
Education level			7.673	0.06
Junior high school	17.86%	45		
senior high school	28.78%	59		
Earthquake-related experiences				
Having been in serious danger (E1)			0.966	0.326
Yes	24.1%	74		
No	20.0%	30		
Having witnessed someone being seriously injured (E2)			24.498	0.000**
Yes	32.88%	72		
No	13.455	32		
Having witness people dying (E3)			10.88	0.001**
Yes	33.91%	39		
No	19.01%	65		
Having close relatives injury severely (E4)			9.641	0.002**
Yes	35.23%	31		
No	19.78%	73		
Having close relatives dead (E5)			0.122	0.727
Yes	20.51%	8		
No	22.97%	96		
Having good friends injured severely or dead E6			6.221	0.013*
Yes	32%	32		
No	20.17%	72		
Having been your body hurt (E7)			3.439	0.064
Yes	32.20%	19		
No	21.36%	85		
Having your house damaged seriously (E8)			10.175	0.001**
Yes	26.3%	91		
No	11.71%	13		
Having witnessed a tragic scene after the earthquake (E9)			10.013	0.002**
Yes	26.59%	88		
No	12.70%	16		
Having felt scared (E10)			13.16	0.001**
Slightly	7.79%	6		
Moderate	22.39%	30		
Intense	27.64%	68		

*P < 0.05, **P < 0.01.

### Relationship Between Personality and PTSD

**Figure 1:**
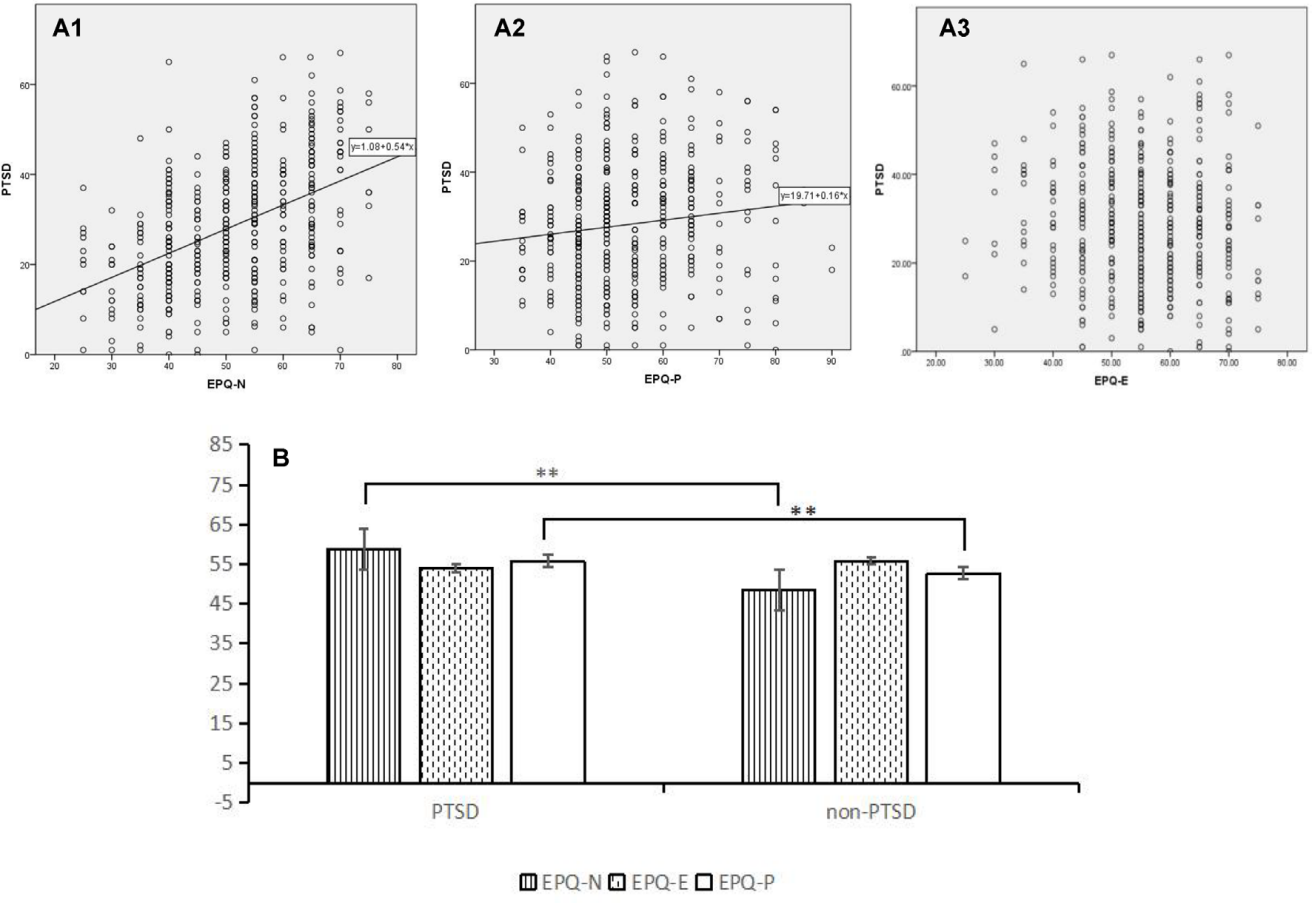
Pearson correlation coefficient analysis of the correlation between the score of EPQ and PTSD. **(A1)** presents the scatter plot distribution between EPQ-N and PTSD; **(A2)** presents that between EPQ-P and PTSD; **(A3)** presents that EPQ-E and PTSD. **(B)** shows means and standard deviations on EPQ scales between PTSD (n = 104) and non-PTSD (n = 353), divided according to the cut-off score of IES-R with the missing values filled with the average. EPQ-N, Eysenck Personality Questionnaires-Neuroticism; EPQ-E, Eysenck Personality Questionnaires-Extraversion; EPQ-P, Eysenck Personality Questionnaires-Psychoticism.

**Figure 1** presents the intercorrelations between personality measured by EPQ and PTSD. EPQ-N had a positive correlation with PTSD (r = 0.454, p < 0.001). Psychoticism was also significantly related to PTSD but not as close as neuroticism (r = 0.128, p = 0.008 < 0.05). To the contrary, there was not a significant relationship between extraversion/introversion and PTSD (r = -.093, p = 0.055 > 0.05). a1 and a2 illustrates the correlation between PTSD and the EPQ-N and EPQ-P respectively, indicating the significant positive linear fit of neuroticism and PTSD (R^2^ = 0.208) but slightly positive linear fit of psychoticism and PTSD (R^2^ = 0.016), whereas a3 shows no significant liner relation between extraversion/introversion and PTSD (R^2^ = 0.009). **Figure 1B** shows the differences of the average standard scores of EPQ between the PTSD group and the non-PTSD group, divided by the same cut-off as **Table 1**, and illustrates the result of t-test, which verified that participants in PTSD group statistically significantly had larger means in the neuroticism assessed by EPQ (PTSD = 58.89 ± 9.07, Not PTSD = 48.5 ± 10.70, t = 8.944, p = 0.000 < 0.01). Extraversion/introversion was not associated with PTSD (PTSD = 54.01 ± 10.20, Not PTSD = 54.84 ± 9.48, t = -1.702 P = 0.09 > 0.05). Psychoticism was significantly associated with PTSD (PTSD = 55.83 ± 10.29, Not PTSD = 52.74 ± 10.39, t = 2.675, p = 0.008 < 0.05). PTSD group had scored higher in neuroticism and psychoticism, but no difference in extraversion/introversion compared with non-PTSD group in our study.

### Analysis of Effects of Traumatic Experience and Personality on PTSD

The results of bivariate logistic regression analysis were elaborated in **Table 2**. Related traumatic experiences, including E2, E3, E4, E6, E8, E9, and E10, and personality, including EPQ-P and EPQ-N were all entered the model. With a forward stepwise method, **Table 2** shows some related traumatic experience enter as independent variables in predicting PTSD, such as E2 (OR = 3.224,CI = 1.816-5.723Wald Value = 15.988), E4(OR = 2.027,CI = 1.077-3.817,Wald Value = 4.791) and E8 (OR = 3.681,CI = 1.695-7.991,Wald Value = 10.852), while others including E3,E6,E9 and E10 were deleted for the model without significant contribution to the prediction. EPQ-N entered the regression model (odds ratio = 1.113, 95% confidence interval = 1.081-1.146, Wald Value = 50.467, P < 0.001) could be a significant predictor to the PTSD, while the EPQ-P was deleted in the model (P > 0.5), meaning that though EPQ-P was related to PTSD but it insignificantly predicted the prevalence of PTSD. Hence, personality and certain special traumatic experience among the individual differences could develop a vulnerability profile for the long-term PTSD.

**Table 2:** Multiple logistic regression using forward stepwise analysis for predicting PTSD among junior and senior high school students following the Wenchuan earthquake, China (N = 457).

	Variables	B	S.E.	Wald	Sig.	OR	95% C.I.	R Square
PTSD								
Step 1	EPQ-N	0.103	0.014	55.449	0	1.109	1.079-1.139	0.254***
	Constant	–6.895	0.804	73.473	0	0.001		
Step 2	E2	1.159	0.283	16.81	0	3.186	1.831-5.544	0.31***
	EPQ-N	0.101	0.014	50.429	0	1.107	1.076-1.138	
	Constant	–7.446	0.854	75.964	0	0.001		
Step 3	E2	1.238	0.291	18.089	0	3.448	1.949-6.099	0.35***
	E8	1.325	0.392	11.422	0.001	3.763	1.745-8.117	
	EPQ-N	0.106	0.015	51.796	0	1.112	1.08-1.144	
	Constant	–8.824	0.998	78.156	0	0		
Step 4	E2	1.171	0.293	15.988	0	3.224	1.816-5.723	0.363***
	E4	0.707	0.323	4.791	0.029	2.027	1.077-3.817	
	E8	1.303	0.396	10.852	0.001	3.681	1.695-7.991	
	EPQ-N	0.107	0.015	50.467	0	1.113	1.081-1.146	
	Constant	–9.004	1.024	77.252	0	0		

PTSD, posttraumatic stress disorder; EPQ-N, Eysenck Personality Questionnaires-Neuroticism SE, standard error; OR, odds ratio; CI, confidence interval; E2-Having witnessed someone being seriously injured; E4- Having close relatives injury severely; E8-Having your house damaged seriously. *P < 0.05, **P < 0.01, ***P < 0.001.

**Figure 2:**
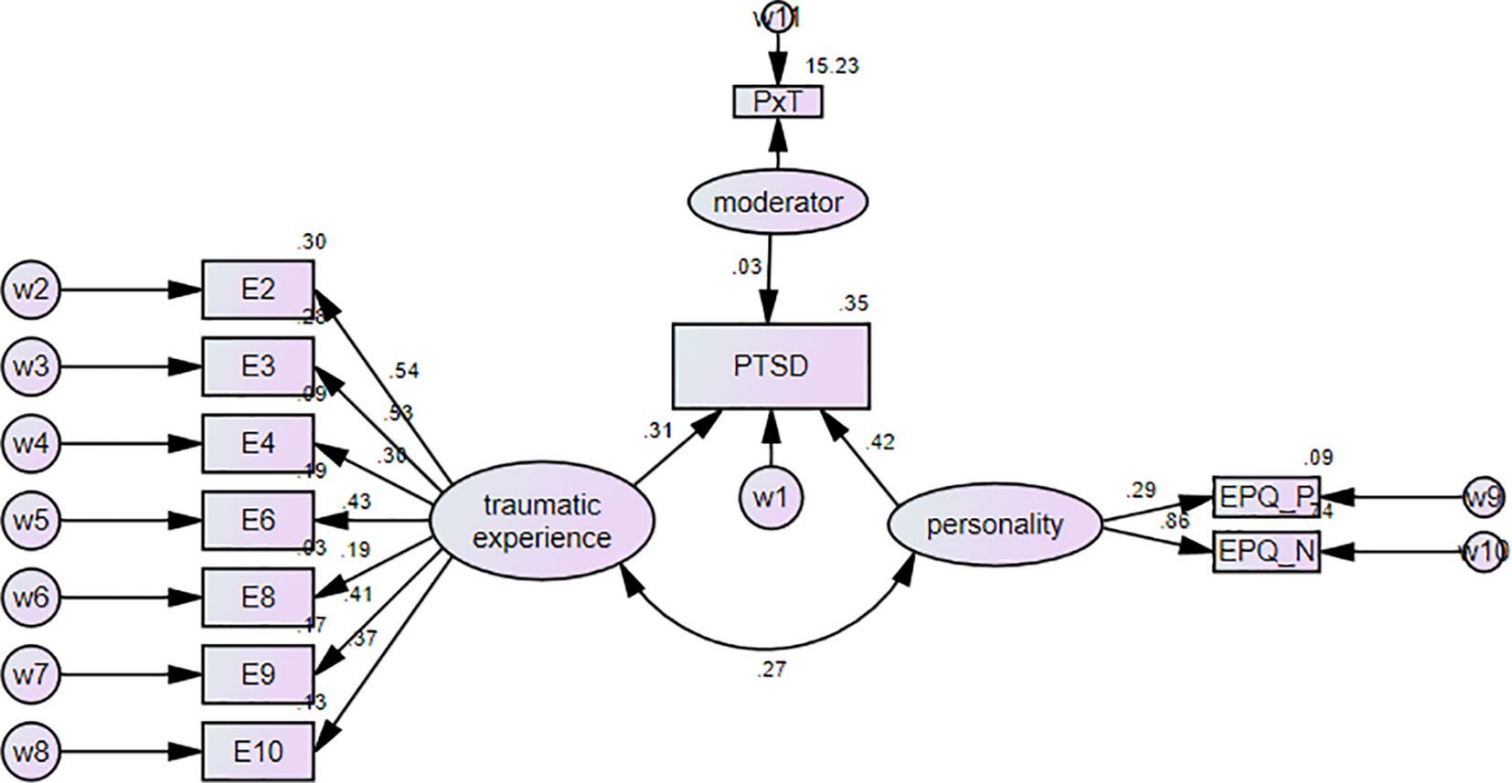
Path diagram of interrelations between predictive factors to PTSD. All the numbers show the standardized regression weight of the variables and all the paths are significant with P value smaller than 0.05. PTSD, posttraumatic stress disorder; EPQ-N, Eysenck Personality Questionnaires-Neuroticism SE; EPQ-P, Eysenck Personality Questionnaires-Psychoticism SE; E2- Having witnessed someone being seriously injured; E3- Having witness people dying; E4-Having close relatives (parents, sisters, brothers, grandparents and so on) injury severely; E5-Having close relatives (parents, sisters, brothers, grandparents and so on) dead in the earthquake; E6- Having good friends injured severely or dead; E7- Having been your body hurt; E8- Having your house damaged seriously; E9- Having witnessed a tragic scene after the earthquake; E10- Having felt scared; moderator is the latent variable loaded with interaction of personality and traumatic experience; PxT- personality interact with traumatic experience; w1-11 are all the residual errors of the model.

The structural model diagrammed in **Figure 2** shows the paths of the factors, which were tested to be related to PTSD in a bivariate logistic regression analysis, linked to the prediction of the severity of PTSD (CMIN/DF = 2.243, P < 0.001; CFI = 0.897 > 0.8; NFI = 0.810 > 0.8; IFI = 0.885 > 0.8; RMSEA = 0.05 < 0.08; P value of every pathway was lower than 0.05). The traumatic experience was significant as an independent latent variable to predicting the PTSD (standard regression weight = 0.31, P < 0.001) and personality as a latent variable was also significant to PTSD (regression weight = 0.42, P < 0.001). Both of them were positively related to PTSD and the correlation between them were also significant (standard regression weight = 0.27, P < 0.001). Notably, the interaction between personality and traumatic experience was significantly related to PTSD (standard regression weight = 0.03, P = 0.012). In our context, the model indicated that people with high scores of personality in the dimension of EPQ-N and EPQ-P had possibly increased severity of PTSD compared with people having the low score in EPQ-N and EPQ-P and the same traumatic experience. Although the standard regression weight of the interaction was relatively low, the weak moderating effect still significantly existed. Besides, with the regression analysis in **Table 2**, the path diagram in **Figure 2**, the significant regression weight in the path demonstrated that EPQ-N, which accounted for a major part of variations in personality (standardized weight = 0.862, P < 0.001), was a stronger predictor for long-term PTSD from a theoretical perspective.

## Discussion

The current study investigated 457 survivors between one and three years after the Wenchuan earthquake. We concluded that the answer for E2, E3, E4, E6, E8, E9, and E10 were closely related to the PTSD. It could imply that the bereavement and interest loss, which hardly compensated for the loss of cherished memories, had the worst influence on someone’s mental health. The result of multivariable logistic regression using backward stepwise elimination showed that E2, E4, and E8 were predictive to PTSD with positive relations, all of which were mental injury caused by a sense of losing the loved, instead of from direct bodily injury. Moreover, personality had a significant relationship with PTSD, which was in line with expectations and previous research. Furthermore, EPQ-N is a significant predictor of PTSD after earthquake. Neuroticism is positively related to PTSD. This finding of personality in the context of earthquake is in line with previous findings in various contexts such as fighters, transportation accidents, industrial and domestic accidents, terrorism, violent crimes, and explosions (29, 41, 42).

Although traumatic experience could be an independent predictor of PTSD (43), the relationship between earthquake trauma, personality and PTSD remains unclear. In a sense, traumatic exposure is a potent environmental risk factor that predicts immediate and lifetime increase in an array of disorder, but it may lead to both negative and positive outcomes. Our results suggested that the earthquake traumatic experience, especially bereavement and deprivation, has a positive relationship with PTSD, implying that the severity of earthquake trauma is related to the severity of mental illness. In addition, we found out the relationship between the latent variables by the way of single product indication, which was an advanced method to testify the moderating effect in latent variables which were measured by limited effective observed variables and contained unobserved elements. The pathway analysis of the structural model indicated that the moderating effect personality has on the relationship between traumatic experiences and PTSD, and 86.1% of variations in personality were accounted for by neuroticism. The moderating role of personality was not strong but significant in our analysis; it deserved further examination by specifying the level of interaction between personality and traumatic experience or adding more effective measurements for unobserved variables. Combining the model with the results of logistic regression analysis, we deem that traumatic experiences like bereavement and deprivation are presumably more likely to elicit PTSD syndromes in neurotic people. Neuroticism, a factor related to the psychological effect of the relief effort and a personality trait characterized by emotional instability and anxiousness, increased susceptibility to psychological trauma. These characters of neuroticism, also identified in studies of Lawrence and Fauerbach (44), increased the arousal symptoms of PTSD, and caused maladaptive coping styles and low mental resilience within neurotic people whilst they had to confront the earthquake trauma (45, 46). From a physiological point of view, personality traits measured by EPQ also influenced the upstream reticular activating threshold in the central nervous system as well as autonomic nervous system functioning (47). People with high levels of neuroticism tended to view the earthquake experiences as more negative and threatening events, reported more daily hassles and stressors in their lives, and experienced more difficulty in coping with stressful situations (25). Therefore, after an earthquake or other traumatic experience, it is necessary to have professional organizations around to urgently assist those who are neurotic with early psychological counseling and social supports to prevent the development of PTSD. In conclusion, the above strategies would help these people to express and stabilize emotion, guide them to calm down, and release the stress which could reduce the prevalence of PTSD.

Although preexisting studies found many factors had a relation to PTSD from the earthquake (15), there was no consistent results for the valid prediction in a long-term perspective considering the complex interactions of these factors. Moreover, our study showed that results were different as the cultural backgrounds varied. For example, we did not replicate the finding that demographic variables (age, gender, ethnicity and education) had a robust relationship with PTSD after the earthquake. It could be assumed that that there might be not an apprehensive sense of social gender roles from the ages of 12 to 20 as the subjects in our study mainly comprised the junior and senior high school students from ethnic minorities who were the major population of the Wenchuan earthquake victims. These people may mature late in the mental state and their gender identity might not be fully formed. However, we thought that junior high school students who were at the stage of incomplete mental development were more vulnerable and deserved more assistance. The education level turned out to be an insignificant factor for predicting PTSD, so we attributed the effect of education levels to the mediating factor of age in accord with the result of Kohn et al. (48). In fact, in our studied cohort, participants had the near-average age and, therefore, we could use age as a controlled variable within the limited range. After all, PTSD research has overwhelmingly relied on retrospective accounts of trauma, which is beleaguered by problems of recall bias. Therefore, neuroticism as a stable variable measured after earthquake would be a plausible predictor for the long-term PTSD.

Some clinical implications could be attained from our study. On the one hand, our results confirmed that personality could be a common factor for mental diseases and neuroticism, especially, was the strongest predictor to PTSD. The defect of personality like high sensitivity and instability should be remedied. In our cohort, these students with PTSD should receive help for the development of personality from the psychological treatment as consummating the personality would be benefit for treating and terminating the chronic PTSD. On the other hand, psychological workers should increase attention and intervention promptly to the neurotic people who experienced trauma in case of the mental injury. Meanwhile, in a sense, self-improvement in personality could be not only praised for self-achievement, but is also good for psychological immunity.

### Limitations and Forwarding Improvements

Several limitations should be noted. First, its cross-sectional design precludes inferences about causality. Second, this study relied entirely on self-report measures. Some researchers suggested that personality could be influenced by traumatic experience, and victims may subsequently perceive themselves, their identity, and reality differently after the experience of earthquake (49). Thus far, the questionnaire of EPQ is thought to measure stable personal traits instead of a temporary coping style. Post-trauma personality measurements may not strictly reflect predisposing vulnerability in trauma victims prior to the traumatic exposure, since we aimed at seeking the effective preventive measures for PTSD after the trauma, so the post-trauma assessment of the personality is more appropriate. However, more information involved in the paper collection and diagnostic clinical interviews would be preferable. Third, some researchers prefer the premise that PTSD is strongly associated with the proximity of the stricken areas or the exposure level to the earthquake rather than with personality 50), which deserves further investigation. However, it is still worth digging into the effect of personality on the rate of PTSD in different traumatic events to see if the exposure level contributes to the prevalence. Lastly, the structural equation model built of the moderating effect of personality is not perfect as the limited indicators for the latent variables, therefore, more significant factors related to the traumatic experience and personality deserve to be attained for testing the moderating effect intensity of personality.

Despite these limitations, we provide stronger evidence to date that personality is correlated to the possibility of PTSD in the survivors experiencing earthquake trauma. Specifically, we identify neuroticism, which implies emotional instability, as a major predictor of the prevalence of PTSD after the earthquake. As post-disaster stressors, traumatic experiences like bereavement and deprivation are also risk factors of PTSD. For the first time, we found that personality has moderating effects on the relationship between earthquake-trauma experience with PTSD in a long term. Intrigued by the Wenchuan earthquake, advanced studies should conduct a systematic investigation for predicting the long-term PTSD and form a more comprehensive understanding of the predictors. These works aim at providing valuable guidance for health care workers to develop efficient early interventions for the trauma population and reduce the prevalence of PTSD. Each year, nature diseases can affect an average of about 200 million Chinese population and kill several thousand people. In the future, this work can be applied to the intervention of PTSD in trauma populations from other nature diseases.

## Conclusion

As one of the largest earthquakes in human history, the devastating Wenchuan earthquake caused tremendous damage in western China. Over the past ten years, people have successfully rebuilt their hometown and secure living environment with the worldwide help. However, many survivors still live in the shadow of mental illness and psychological distress, so the rebuilding of a spiritual home deserves continuing efforts. Our study hopes to commemorate the victims to contribute to the recovery of mental health among the injured survivors. We found a close relationship between personality and PTSD after earthquake, and personality moderate the effects of traumatic experience on PTSD. Specifically, neuroticism might be a stable vulnerability to the PTSD in the long term. Therefore, neurotic people who are exposed to traumatic experiences of bereavement or deprivation should receive more attention and early health interventions.

## Author Contributions

QY was the first author responsible for the writing and data analysis. WL was the equal first author for the review and data analysis. XY contributed to the edition and review. LW was the director of the research and the corresponding author.

## Funding

This research was supported in part by a grant from Shanghai Pujiang Program (13PJC003) and Innovation Program of Shanghai Municipal Education Commission (14ZS084).

## Conflict of Interest Statement

The authors declare that the research was conducted in the absence of any commercial or financial relationships that could be construed as a potential conflict of interest.
